# Taxonomy based analysis of force exchanges during object grasping and manipulation

**DOI:** 10.1371/journal.pone.0178185

**Published:** 2017-05-31

**Authors:** Sandra Martin-Brevet, Nathanaël Jarrassé, Etienne Burdet, Agnès Roby-Brami

**Affiliations:** 1 Sorbonne Universités, Université Pierre et Marie Curie Paris 06, Paris, France; 2 CNRS, UMR 7222, Institut des Systèmes Intelligents et de Robotique, Paris, France; 3 INSERM, U1150, Agathe-Institut des Systèmes Intelligents et de Robotique, Paris, France; 4 Imperial College of Science, Technology and Medicine, London, United Kingdom; 5 Nanyang Technological University, Singapore, Singapore; University of Exeter, UNITED KINGDOM

## Abstract

The flexibility of the human hand in object manipulation is essential for daily life activities, but remains relatively little explored with quantitative methods. On the one hand, recent taxonomies describe qualitatively the classes of hand postures for object grasping and manipulation. On the other hand, the quantitative analysis of hand function has been generally restricted to precision grip (with thumb and index opposition) during lifting tasks. The aim of the present study is to fill the gap between these two kinds of descriptions, by investigating quantitatively the forces exerted by the hand on an instrumented object in a set of representative manipulation tasks. The object was a parallelepiped object able to measure the force exerted on the six faces and its acceleration. The grasping force was estimated from the lateral force and the unloading force from the bottom force. The protocol included eleven tasks with complementary constraints inspired by recent taxonomies: four tasks corresponding to lifting and holding the object with different grasp configurations, and seven to manipulating the object (rotation around each of its axis and translation). The grasping and unloading forces and object rotations were measured during the five phases of the actions: unloading, lifting, holding or manipulation, preparation to deposit, and deposit. The results confirm the tight regulation between grasping and unloading forces during lifting, and extend this to the deposit phase. In addition, they provide a precise description of the regulation of force exchanges during various manipulation tasks spanning representative actions of daily life. The timing of manipulation showed both sequential and overlapping organization of the different sub-actions, and micro-errors could be detected. This phenomenological study confirms the feasibility of using an instrumented object to investigate complex manipulative behavior in humans. This protocol will be used in the future to investigate upper-limb dexterity in patients with sensory-motor impairments.

## Introduction

Hand dexterity is a major attribute of the human species, and the ability to manipulate objects is probably at the basis of evolution of tool use and cognition [1]. Dexterity may be defined as “finding a motor solution for any situation and in any condition” [2, 3]. The highest degree of dexterity in animals belongs to the human hand, which is particularly versatile with many degrees of freedom allowing a wide range of sophisticated interactions with objects. The precise analysis of finger movements [4, 5] requires a precise laboratory set-up, it is thus difficult to investigate voluntary movements in ecological contexts such as ergonomics and clinical applications. However our hypothesis is that the dexterity to manipulate an object can be quantified from this object motion and the force exchanged between the hand and the object. Therefore we developed an instrumented object providing measurement of motion and force on all its faces [6] to examine a variety of grasping and manipulation tasks representative of object use in the daily life [7]. Towards this ambitious goal, we analyze here the exchange of forces during holding and manipulation tasks of scaled difficulties. While previous studies provided in depth analysis of precision grip [8], we investigate here a wider variety of grasping postures selected from two recent taxonomies of grasping and manipulation [3, 9].

### Qualitative taxonomies of hand function

Napier [10] was the first to identify two main classes of prehensile hand postures according to the task’s demand and fingers’ configuration. The *precision grip* is a multi-pulpar opposition between fingers and the adducted thumb used for tasks requiring precision. *Power grasp*, where the object is held between fingers and palm reinforced by the abducted thumb, is used for power tasks involving a high level of force. Iberall et al. [11] later proposed a systematic description according to the direction of the forces applied by the fingers by reference to the three axes of the palm, and identified an intermediate class: lateral opposition as for holding a key. They also defined virtual fingers as units of one or several fingers (or palm) working in opposition to form the grasp [11]. Based on these concepts, Feix et al. [9] proposed a systematic taxonomy of grasping, and synthesized the examples found in the literature in 33 grasp types or 17 more general classes. A grasp is defined as *“every hand posture with which an object can be held securely with one hand*, *irrespective of the hand orientation*” [9] and thus involves at least two virtual fingers [12].

In addition to grasping, manipulation requires the ability to move the object within the hand using independent fingers movements [13, 14]. Bullock and Dollar [3, 12] proposed a systematic, hand centered classification of manipulation tasks according to the direction of the movement of the object relative to the hand. The following criteria were used to classify these tasks: existence of contact (with the object or the environment), existence of prehensile posture, motion of the hand relative to the body, motion of the fingers relative to the hand, motion of the object relative to the contact points on the hand. A study in an ecological professional context (household and machinist) showed that individuals used only few different manipulation patterns during their activity [15].

### Force exchanges during grasping

A milestone in the study of manipulation was provided by the pioneering work of Johansson and colleagues [16], who studied the evolution of grip and load forces during lifting with a precision grip. They observed that grip forces increase in parallel to load forces without time lag, demonstrating anticipation in the fingers control, then yield a steady level with a safety margin during holding [16]. The relationship between grip and load forces control was modulated as a function of the object physical properties, such as weight [17], contact surface [18], size [19], and shape [20]. The anticipation of grip force as a function of load force was also observed in experiments where the object is transported [21–23], grasped by a part away from the centre of mass [24–26] or rotated [27, 28]. The regulation of grip force and finger positions is anticipated as a function of the predicted dynamics of the interaction between the object and the environment in order to ensure stable interaction with the object or minimize its deviation [24, 25, 29, 30]. The anticipatory control is based on both generic representations of the object properties acquired during prior manipulative actions as well as specific context memories [24, 25, 31, 32]. A few trials are sufficient to develop an anticipatory behavior [33, 34]. In addition, the grip force is finely regulated by the feedback of the tactile afferents [23, 35]. Multisensory afferent feedback elicited by the events and mechanical transients during the lifting task is used to monitor and control the sequential execution of the series of phases or sub-goals during the manipulative action [35].

However, all these experimental studies focused on the precision grip for grasping relatively small objects picked between index and thumb. Other studies examined the force exerted by individual fingers for lifting and turning an object (see review in [5]). They demonstrated that the stability of the object was provided by force sharing between fingers allowing slip prevention, tilt prevention and resistance to perturbations [36]. Furthermore, these studies allow examining the sensorimotor control of precision grip by the central nervous system, but they can hardly be generalized to the prehension and manipulation of objects during natural tasks in daily life. Most manipulanda were designed for research purposes in healthy subjects except one developed for patients with neurological impairments [37]. These instrumented objects do not induce very diverse compensatory grasping strategies [38] or complex manipulative behaviors. [5] and can hardly be used to examine how motor strategies are modified with neurological impairments.

### Objectives

The aim of the present study is to fill the gap between qualitative taxonomies of manipulative behavior and quantitative analyses of force exchange between the hand and an object. In this purpose, we selected a set of tasks inspired by Bullock’s and Feix’s taxonomies [3, 9], which are representative of tasks of daily life, and demand diverse reach directions and hand orientations [39]. The longer term objective is to establish a pertinent methodology for the analysis of grasping in pathological situations where patients with impaired hand control can develop various compensatory strategies [38].

## Materials and methods

### Apparatus

**Fig 1:**
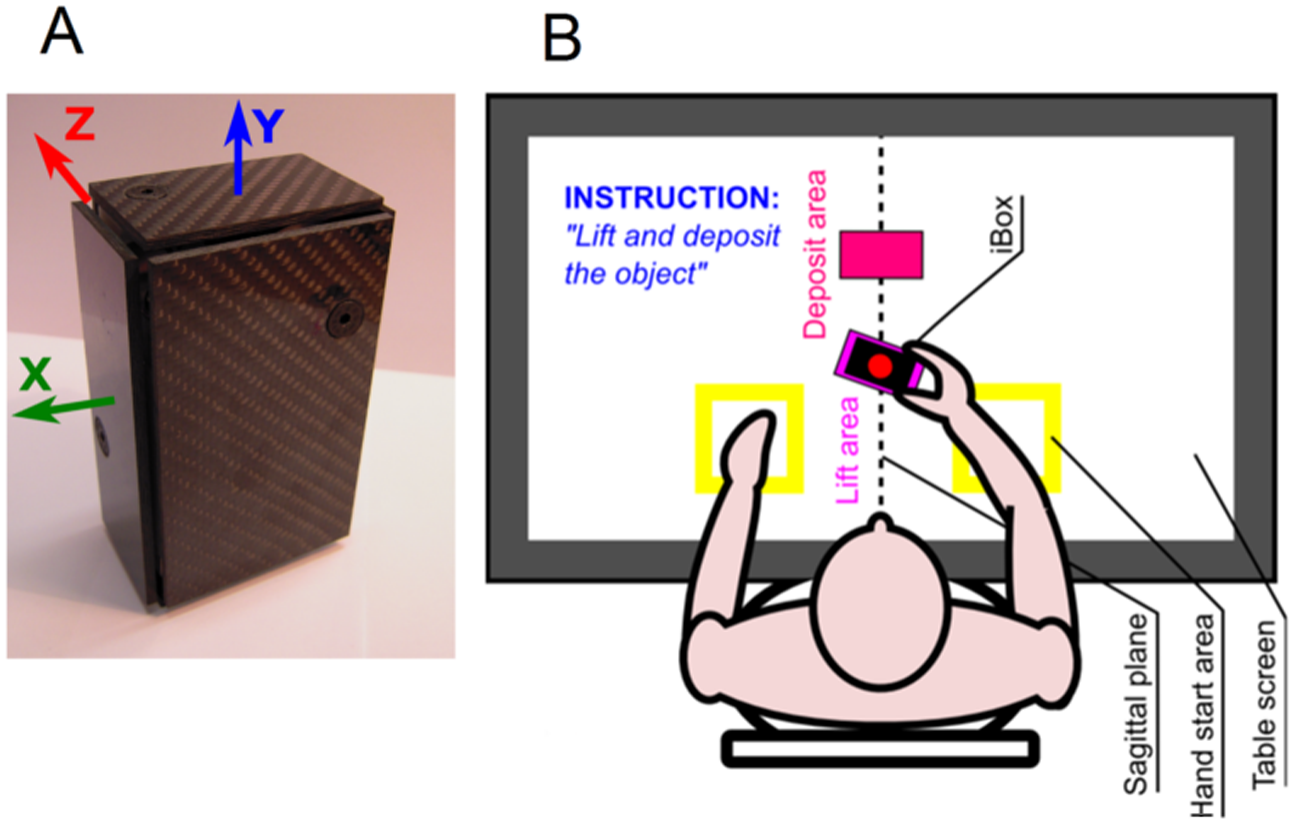
Schema of the iBox (A), and experimental set-up (B).

The iBox [6] is a parallelepiped (Fig 1A) of dimension 108x70x40mm^3^ and mass 0.370kg. It is equipped with an inertial unit (IMU), an embedded electronic board [40] to measure its accelerations, rotational velocities, orientations and six load cells to measure the force applied normally to its six faces (up to 42 N). The IMU and force signals are time stamped and transmitted wirelessly through bluetooth to a distant computer at a 100Hz frequency, with a computer program to monitor the acquisition of data triggered and stopped by the experimenter. The values of the IMU and force signals are initialized while the iBox is put down on the table so that the bottom face, on which the object laid, measures a negative force of 3.63N when the object is lifted.

The sensitivity (experimentally assessed) of the force measurement is 0.01N with a range of 0.01-42N and an accuracy of 1.5%. However, the measure varies with the point of application of the force relative to the fixed base of the sensor, due to a torsional deformation. This error is therefore higher on the “large” front/rear faces (8% for the maximum distance, 5% in the central zone) and below 2% for the lateral and top/bottom faces. The inverse arrangements of the sensors can induce asymmetries in the opposite faces, that are minimal in the central zone.

### Subjects

24 healthy subjects between 20 to 31 years old, with 10 females, participated to this study. All reported to be right handed, although 4 were ambidextrous according to Edinburgh questionnaire [41]. Their hand’s size measured between the tips of major finger and the wrist crease varied between 17 and 20cm for males and 16 and 18cm for females. The experiments were performed in accordance to the principles of Helsinski Declaration and approved by the Ethic Committee at University Paris Descartes. All subjects signed an informed consent prior to starting the experiment.

### Installation

Each subject was sitting on an adjustable chair in front of a horizontal table with a visual screen display placed approximately at the level of the navel (Fig 1B). The initial posture was with both hands laying on the edge of the table, at a comfortable distance. The initial position of the iBox was placed in the sagittal plane of the subject at a standardized distance (80% of his arm length measured from the acromion to the metacarpo-phalangeal joint of the medius). For all the tasks, the iBox was presented with its main axis Y vertical (see Fig 1A), with the initial basis position indicated by a rectangle on the table screen, rotated of -30° by reference to the frontal plane (60° for the power grasp) in order to ensure a comfortable grasp [38]. In this initial position, the faces of the iBox were defined as front/rear (the largest faces), Left/Right and Top/Bottom. The screen of the table was also used to indicate the deposit place for the transport task.

### Tasks description

**Fig 2:**
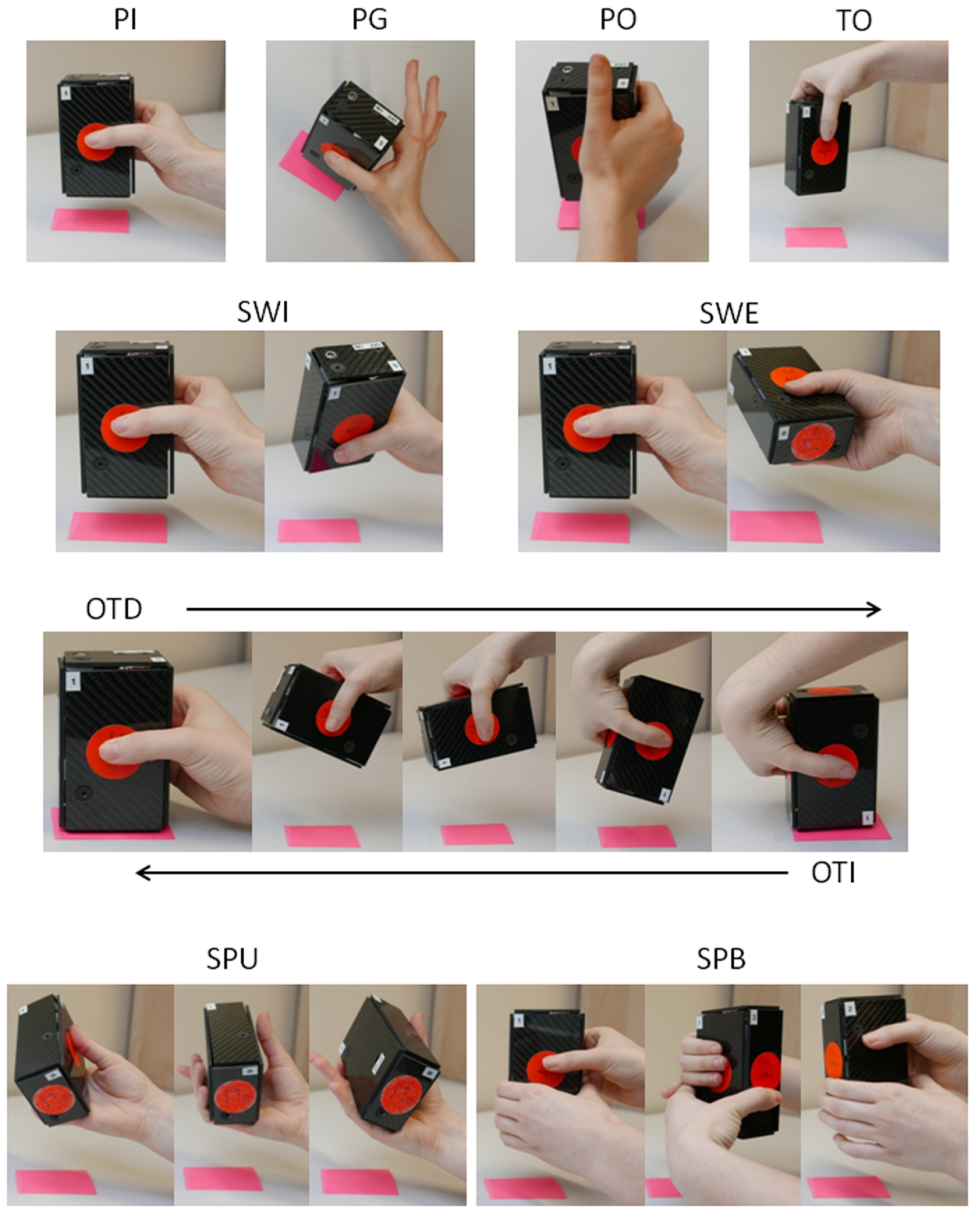
Schema of the holding and turning tasks. PG: Precision Grip, PI: Pinch Grip; PO: Power Grasp, TO: Top Grasp and Move, SWI: Internal Swing, SWE: External Swing, OTD—OTI: Direct or Indirect Overturn according to the direction of the arrow; SPU: Unimanual Spinning, SPB: Bimanual Spinning.

The tasks were chosen and the instructions specified after some preliminary tests with nine subjects. The eleven tasks (ten of which are illustrated in Fig 2) combine different types of grip, actions of the hand and movements of object relative to the body and to the hand.

For the **holding tasks**, the subjects has to lift the iBox approximately 10cm above the table [42] then either to hold it around 2s before replacing it approximately on its initial position, or to put it on a distant target at 10 cm on the screen for the fourth task, oriented parallel to the frontal plane. The following grasping configurations are requested:

Pinch Grip (PI): opposition between the pad of thumb and the ventral face of the other fingers.

Precision Grip (PG): opposition between the pads of thumb and index.

Power Grasp (PO): opposition of fingers and palm, with the thumb in abduction as for a power task.

Top Grasp and move (TO): approach of iBox from the top, opposition with a pinch grip and deposit of iBox on a constraint position in the frontal plane.

For the **translation task** (TR) the subjects has to lift the iBox using the pinch grip then to translate the iBox inside their hand towards a power grasp with thumb in adduction.

For the **turning tasks**, the subjects has to lift the iBox, turn it around the requested axis then to replace it approximately on its initial position.

Swing: the instruction is to rotate the iBox around the X axis approximately 90° internally (SWI: *Internal swing* or “*pretend to drink*”) or externally (SWE: *External swing* or “*pretend to read something written on the bottom of the object*”). During SWE, the iBox is grasped mainly between the thumb and the lateral face of the index.

Overturn: the instruction is to rotate the iBox around the Z axis and to place it upside-down. *Direct overturn* (OTD, 180° anticlockwise) begins with the thumb pointing up and *indirect overturn* (OTI, 180° clockwise), from the left side, with the thumb pointing down.

Spinning: the instruction is to rotate the iBox 360° around the Y axis in the clockwise direction using either one hand (SPU, *Uni-manual spinning)* or alternatively two hands for each 90° step (SPB, *Bimanual spinning*).

In brief, these tasks impose rotation around the vertical axis (Spinning tasks and Top Grasp and Move), an axis parallel to the opposition axis (Overturn) and an axis grossly parallel to the forearm axis (Swing).

### Experimental procedure

Each subject began by several unconstrained lifting tasks with a spontaneous hand configuration to become familiar with the iBox weight and surface characteristics. Then the subjects performed the 11 tasks in a random order. The instructions were given on a written document with pictures on the table screen, supplemented with verbal explanations if needed but the subjects were not allowed to practice before recording. The subjects had to perform the task three times with their right hand (or beginning by the right hand for the bimanual task). The experimenter regularly checked the initial posture of the subjects and repositioned the iBox on its initial position if needed between movements.

The dataset supporting the conclusions of this article is available in the Open Science Framework repository (https://osf.io/pq5jn/).

### Data analysis and statistics

**Fig 3:**
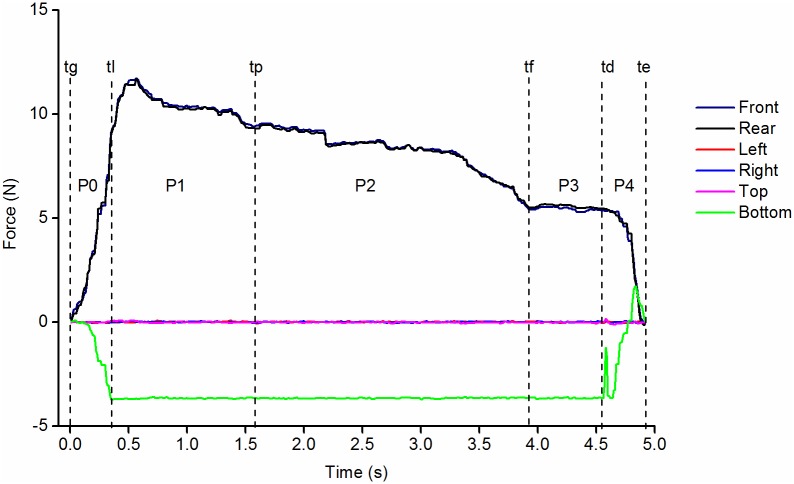
Presentation of the typical times and phases. Example of the forces applied on the faces of the object during Pinch Grip. The forces on the front and rear faces (black) are positive during grasping. The force on the bottom of the object (green) is negative when unloaded, the forces on the lateral and top faces are null. The vertical stippled lines represent the typical times which separate the different phases of the action: P0: unloading (between tg, onset of grasping and tl, onset of lifting); P1: lifting (between tl and tp); P2: holding (between tp, end of peak force and tf, end of plateau); P3: preparation to deposit (between tf and td, end of lifting) and P4: deposit (between td and te, end of grasping).

The analysis focused on the forces applied on the 6 faces of the iBox (Fig 3), on the angles computed from sensor fusion of the IMU signals [6] and on global acceleration (computed as the norm of the three vectors). The rotations were presented as Euler angles in YXZ sequence (corresponding respectively to spinning, swing and overturn). Data analysis was performed with a dedicated Matlab script.

The following typical times were automatically determined from the force signals (Fig 3). The *onset of grasping* (*tg*) which is the time when the sum of the forces on all the faces reached 0.15N, the *onset of lifting* (*tl*), when the bottom force sensor is unloaded (when its force signal decreased below the threshold of -3.4N), the *onset of deposit* (*td*) characterized by the loading on the bottom face of the object (when the force increased from -3.63N to a threshold of -3.4N), and the *end of grasping* (*te*) is when the sum of the forces on all the faces becomes less than 0.15N.

For the turning tasks, the *beginning* (*tp*) and *end* (*tf*) *of the manipulation period* are determined from the angle signals: ± 25° around the X axis for swing, ± 25° around the Z-axis for *tp* and ± 165° for *tf* for overturn. For the spinning tasks, *tp* is the time when the sum of the forces on the right and left faces exceeded that on the front and rear faces, conversely *tf* is when the sum of the forces on the front and rear faces exceeded that on the right and left faces. For the other tasks, *tp* and *tf* were manually tagged: *tp* after the initial force peak and *tf* at the approximate end of a force plateau for the holding tasks or just after the brisk variation of forces indicating translation.

These times separate the five phases of action: *unloading* (P0) is between *tg* and *tl*, *lifting* (P1) between *tl* and *tp*, *holding* or *manipulating* (P2) between *tp* and *tf*, *preparation to deposit* (P3) between *tf* and *td*, and *deposit* (P4) between *td* and *te*.

The occurrence of downward “*pushes”* on the object were detected by a positive force (>0.4 N) on the bottom face during unloading or deposit phases. The amount of force applied to all the object’s faces during the 100ms preceding grasping (before *tg*) and following the release of the object (after *te*) was also calculated. A “*touch”* of the iBox was noted when this cumulated force was greater than 0.7 N.

The variables that were analyzed were:

- The forces applied on each face at the typical times *tg*, *tl*, *td*, *te*.
- The duration of the unloading (P0) and deposit (P4) phases.
- The rate of force increase during P0 (force at *tl* divided by the duration of P0) and of force decrease during P4 (force at *td* divided by the duration of P4)
- The orientation of the object at the typical times *tg*, *tl*, *td*, *te*.
- The median of the forces applied on each face during P1, P2 and P3 phases.
- The occurrences of downward *“pushes*” on the object before lifting or during deposit and of *“touche*s” before grasping or after object release.

Since the force variables was found to be not normality distributed, statistics were performed using non parametric tests (Kruskall Wallis or Mann-Whitney for tasks comparison and Wilcoxon for paired comparison), and the results were presented as median and inter-quartile [median (IQ)] of 72 trials (3 repetitions made by 24 subjects).

The signals were synchronized on the time of lifting (tl) and averaged from -150 ms before to 450ms after tl. Averages were performed over the subjects and repetitions for each task.

## Results

**Fig 4:**
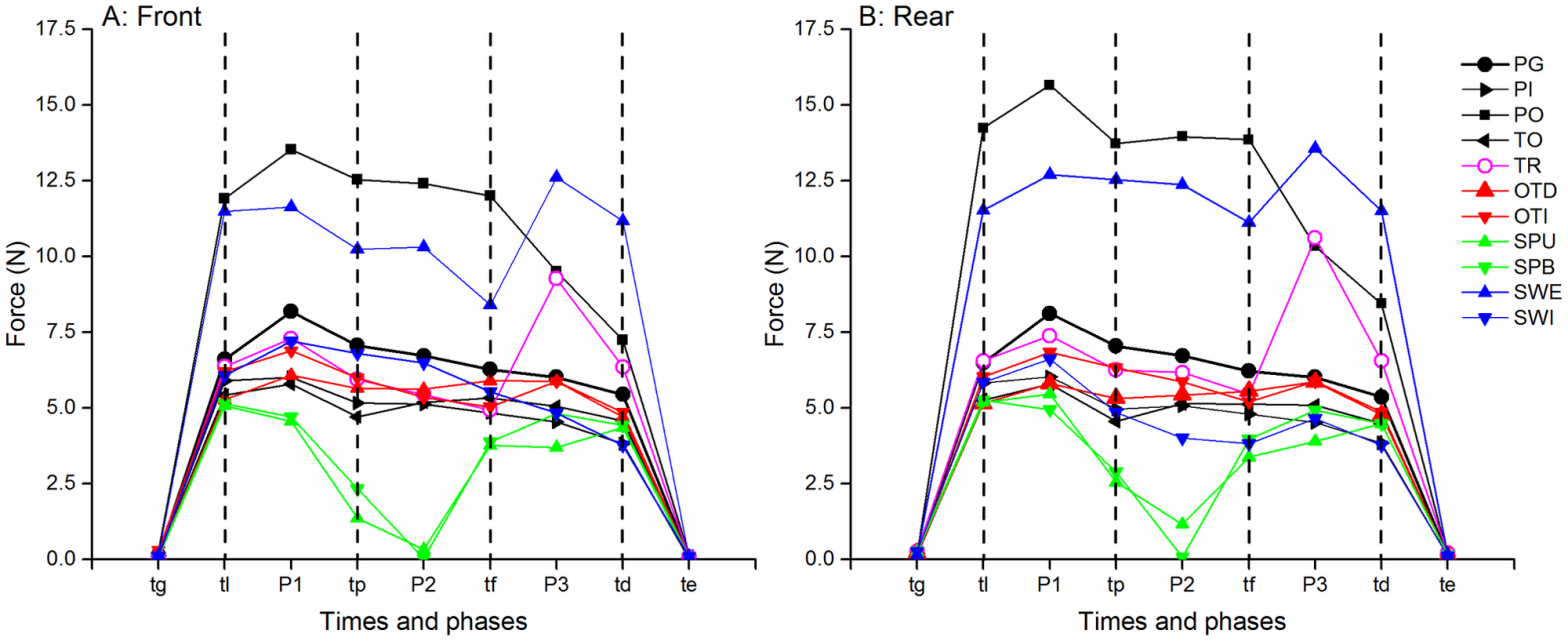
Median forces observed during the different tasks. The front face is illustrated on the left and the rear on the right. The vertical lines correspond to the typical times (same legend as Fig 3) that separate the phases of action. Each point represents the median of 3 repetitions in 24 subjects (median were calculated over values at typical times or over median of the values during P1-P3 phases). The black symbols represent lifting tasks (PG: precision grip, PI: Pinch; PO: Power; TO: Top grasp) and color symbols translation (TR) and turning tasks (SWI: internal swing, SWE: external swing, OTD Direct overturn, OTI: indirect overturn; SPU: unimanual spinning, SPB: bimanual spinning).

The evolution of force signals during the different tasks generally showed a similar pattern in 5 phases. The level of forces during the different phases varies with the task (Fig 4). The results will be presented successively for the different phases: unloading (P0), holding or manipulation (P1-P3) then deposit (P4).

### Unloading phase (P0)

The unloading phase (P0) was characterized by an increase of grasping forces on the front and rear faces of the iBox while the force on the bottom face decreased until it reached the threshold of -3.4N at *tl*, indicating complete unloading of the object.

Grasping was sometimes preceded by sub-threshold *“touches”* of the object, with a frequency that varied with the task (from 5.6 to 19.4%, Table 1). Lifting was sometimes preceded by a downward “*push*” on the object, manifested by a positive force on the bottom face. The frequency of apparition of *“pushes”* varied with the task from 2.8 to 42.3% (Table 1).

**Table 1:** Frequency of “*touches*” before grasping and downward “*pushes*” during P0.

	Touches	Pushes	
Tasks	%	%	Force (N)
Precision Grip	8.3	2.8	0.5
Pinch Grip	5.6	5.6	0.5
Power Grasp	19.4	37.5	0.6
Top Grasp and move	9.7	16.7	0.6
Translation	8.7	11.6	0.5
External Swing	8.4	42.3	0.9
Internal Swing	14.3	25.4	0.6
Direct Overturn	8.3	23.6	0.9
Indirect Overturn	11.1	38.9	0.8
Unimanual Spinning	17.7	11.3	0.5
Bimanual Spinning	10.5	14	0.7

**Fig 5:**
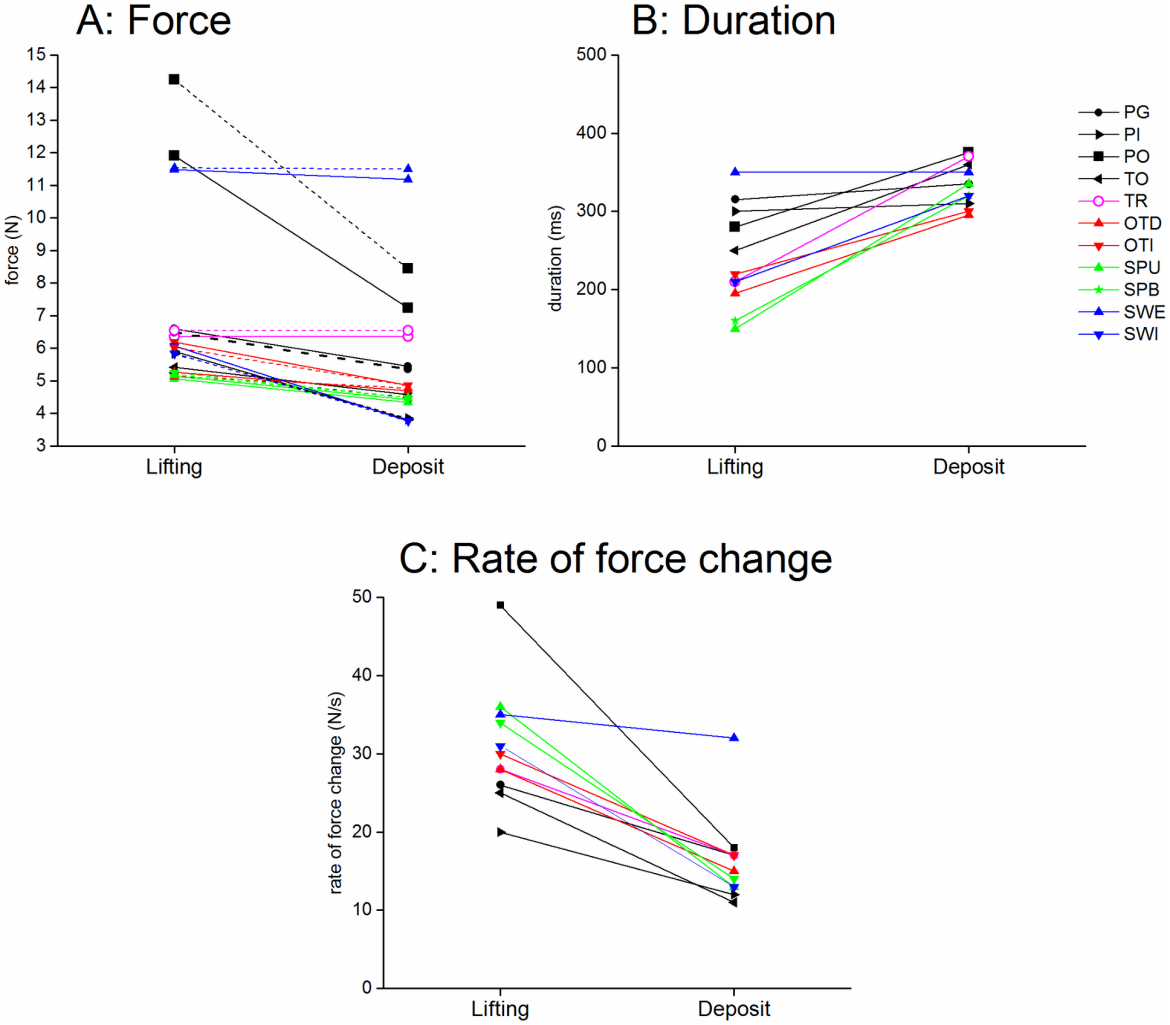
Comparison of unloading and deposit phases. A: Forces applied to the front and rear faces at the times of lifting (tl) and deposit (td). Each line represents the median values of 3 repetitions in 24 subjects for the given task (see legend). The forces on the front and rear faces are represented by a full and a dashed line respectively. B: Duration of the lifting (P0) and deposit phases (P4). Same legend. C: Rate of force change during the unloading and deposit phases.

The grasping force measured at *tl* (Fig 5) varied significantly with the task for both the front and the rear faces (Kruskal-Wallis, p<0.0001). One-by-one Wilcoxon tests confirmed that the force level was maximal for Power Grasp and External Swing and lowest for Spinning and Top Grasp and move. The duration of the unloading phase also varied significantly with the task (Kruskal-Wallis, p<0.0001) as well as with the rate of force increase (Kruskal-Wallis, p<0.0001). The forces applied on the right face of the object remained close to zero (less than 0.05N) except for power grasp (1.46N (1.5), median and interquartile) and external swing (0.42N, (1.25)). The forces applied on the left and top faces were always close to zero, (excepted on the left face for indirect overturn). The level of force was generally slightly different on the rear and front faces.

The grasping force measured at *tl* (Fig 5) varied significantly with the task for both the front and the rear faces (Kruskal-Wallis, p<0.0001). One-by-one Wilcoxon tests confirmed that the force level was maximal for Power Grasp and External Swing and lowest for Spinning and Top Grasp and move. The duration of the unloading phase also varied significantly with the task (Kruskal-Wallis, p<0.0001) as well as with the rate of force increase (Kruskal-Wallis, p<0.0001). The forces applied on the right face of the object remained close to zero (less than 0.05N) except for power grasp (1.46N (1.5), median and interquartile) and external swing (0.42N, (1.25)). The forces applied on the left and top faces were always close to zero, (excepted on the left face for indirect overturn). The level of force was generally slightly different on the rear and front faces.

There was no modification of the orientation of the object by reference to the vertical during P0, the median angles around X and Z remaining less than 0.5° at the onset of lifting.

### Lifting phase (P1)

The lifting period was characterized by a force peak: the grip forces continued to increase significantly after lift-off then decreased significantly to reach a lower level during P2. A force peak was observed in all the tasks with the exception of spinning.

**Fig 6:**
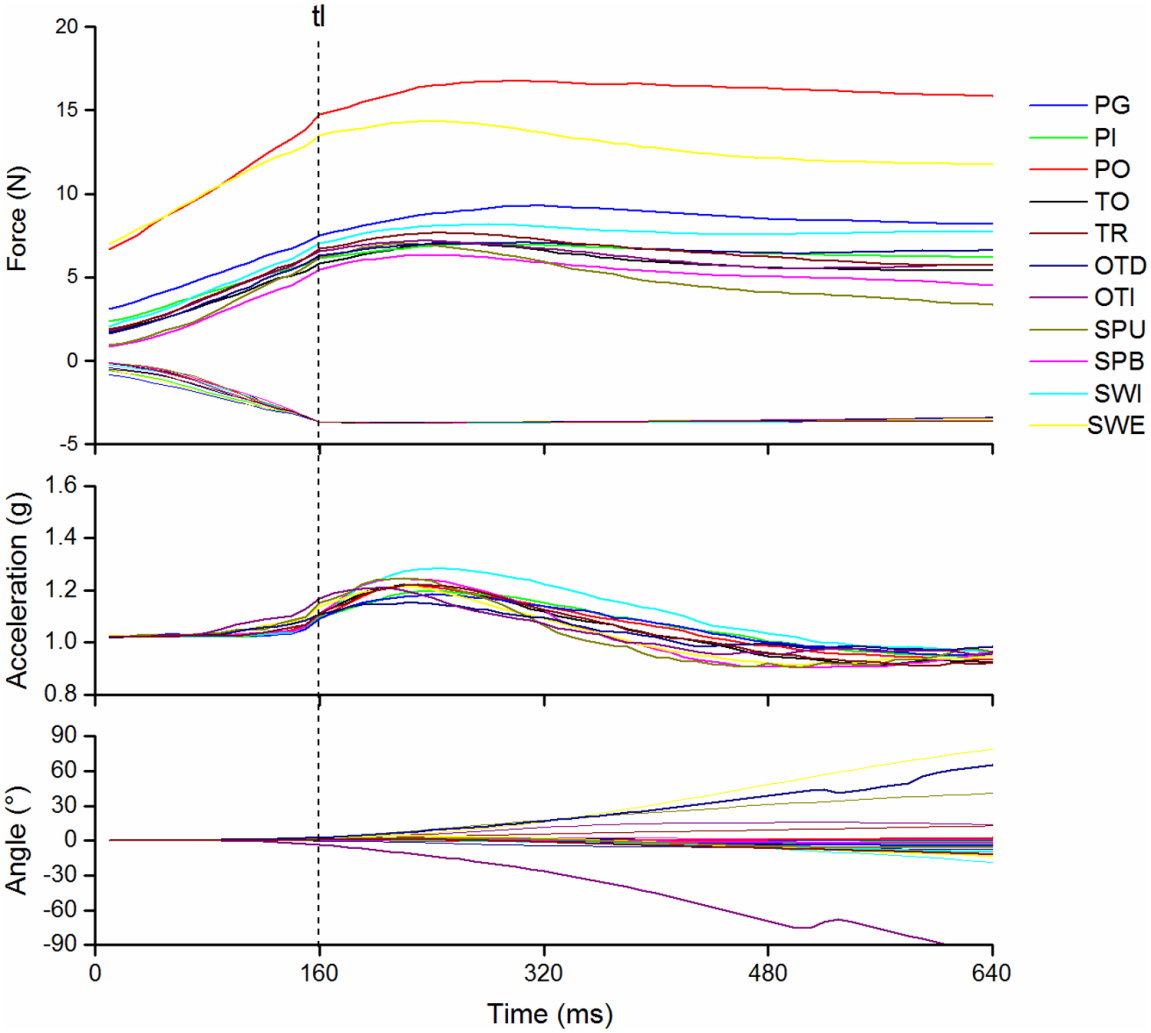
Lifting phase for the different tasks. Averages of forces, acceleration and angle signals performed across participants and trials after synchronization on the time of lifting (tl).

The timing of events during lifting is illustrated by averages presented on Fig 6. The force peak was at the same time as acceleration followed by a progressive deceleration. The angles around X and Z changed mainly during the deceleration period of lifting. However, the timing of the lifting movement could not be specified on a trial-by-trial basis due to noisy acceleration signals in the absence of external precise motion capture.

### Holding or manipulation phases (P2-P3)

#### Holding tasks

**Fig 7:**
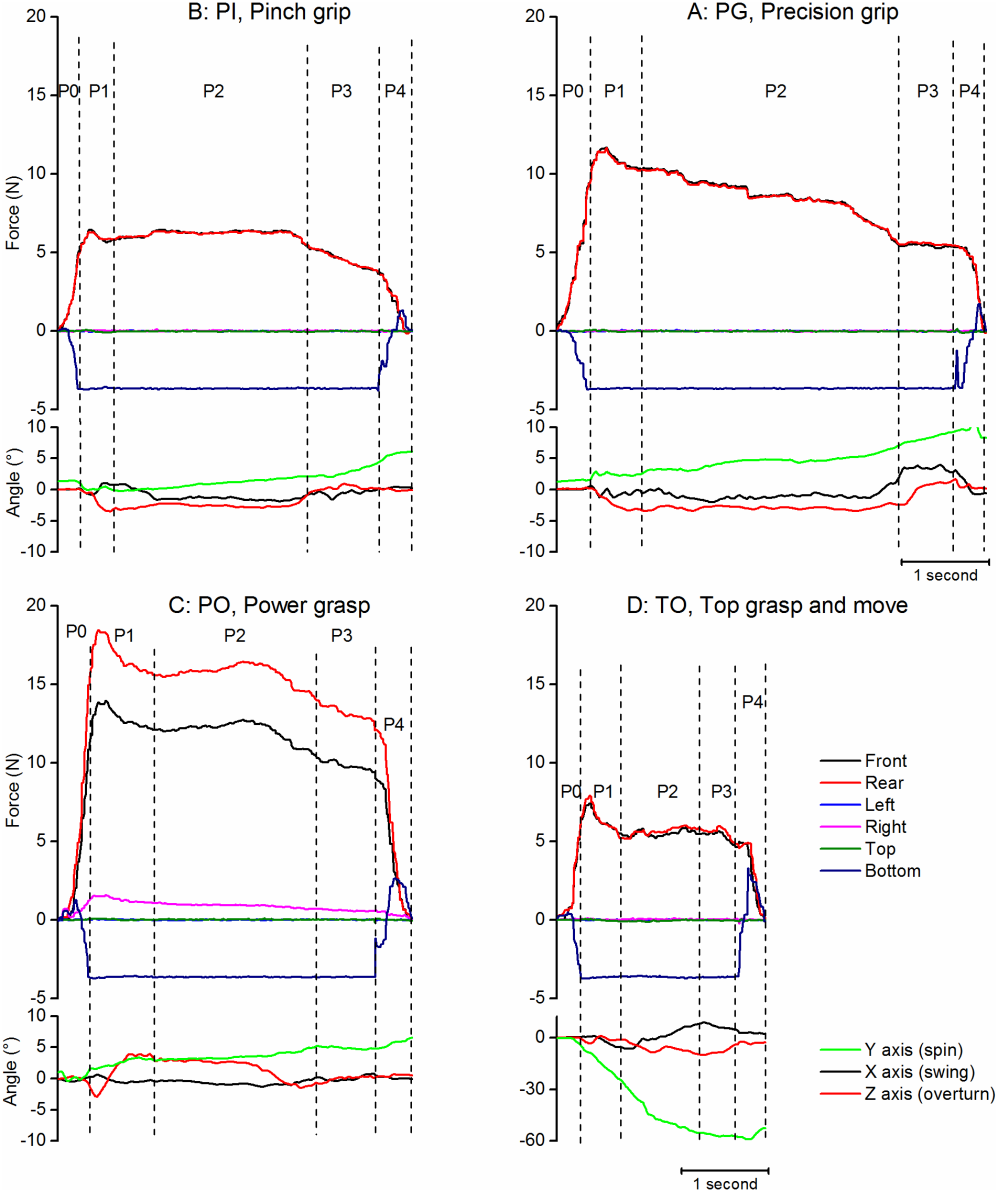
Examples of holding tasks. A: Precision Grip (PG), B: Pinch Grip (PI), C: Power Grasp (PO), D: Top Grasp and move (TO). Each figure represents one trial in the same representative subject. The upper part of each figure represents the evolution of the time forces on the faces of the object and the lower part the variations in the orientation of the object according to the 3 axis (see color legends in D). Phases and vertical stippled lines: same legend as Fig 4.

During holding tasks (Fig 7), the intermediate period (P2) corresponded to a plateau with a relative stability of the forces on the front and rear faces, or sometimes a progressive increase and decrease as a dome for Power Grasp. The median force on the front and rear faces varied significantly with the task (Kruskall Wallis, p<0.0001). One to one comparison with the Wilcoxon test showed that the forces were small during Pinch Grip (5.1N (3.2) and 5.0N (3.2) respectively for the front and rear faces, median and interquartile) and Top Grasp and move (5.1N (2.7) and 5.1N (2.7)). Conversely, the forces were maximum with a great variability during Power Grasp (12.4N (12.8) and 13.9N (13.0)). They were intermediate during Precision Grip (6.7N (3.2) and 6.7N (3.4)). The negative force on the bottom face -3.63N (0.04) corresponded to the unloaded weight of the object. The forces applied on the other faces of the object remained close to zero, except on the right face for Power grasp 1.4N (1.2) and sometimes (in 12/72 trials) on the left face during Top Grasp.

The object remained close to vertical during P2 (with less than 3° deviation around the X and Z axes). The axial orientation of the object (around Y) varied during TO task, according to the instruction to place the object with another orientation: it was -25° (20.6) at the beginning and -49.5° (13.3) at the end of P2.

The preparation to deposit (P3) was characterized by a progressive decrease of grip forces with significant difference between P2 and P3 (Wilcoxon p<0.01 to p<0.0001).

#### Translation task

**Fig 8:**
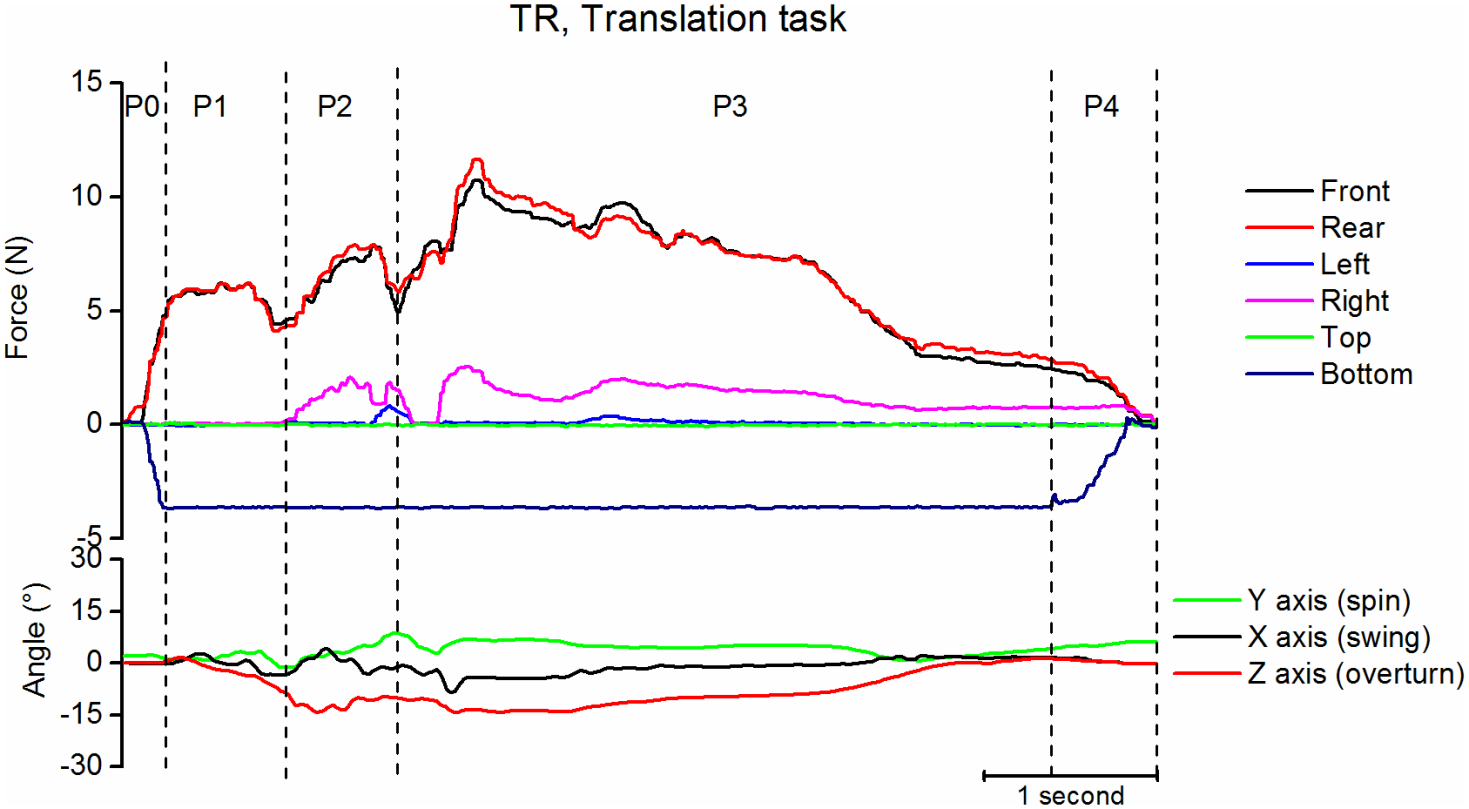
Example of translation task. Same legend as Fig 7.

For the translation task (Fig 8) the object had to be passed from Pinch Grip to Power Grasp. The beginning of the task was similar to Pinch Grip (with a similar level of force on the front and rear faces during P2 (5.4N (3.6) and 6.1N (3.0)). The time of translation was indicated by a brisk decrease of force (but its amplitude was not quantified) followed by a secondary force increase involving also the right/left faces of the iBox. The later period of the task (P3) was similar to Power Grasp with similar levels of force on the front and rear faces (9.2N (6.9) and 10.6N (6.0) and on the right face (2.5N (2.3)).

#### Swing tasks

**Fig 9:**
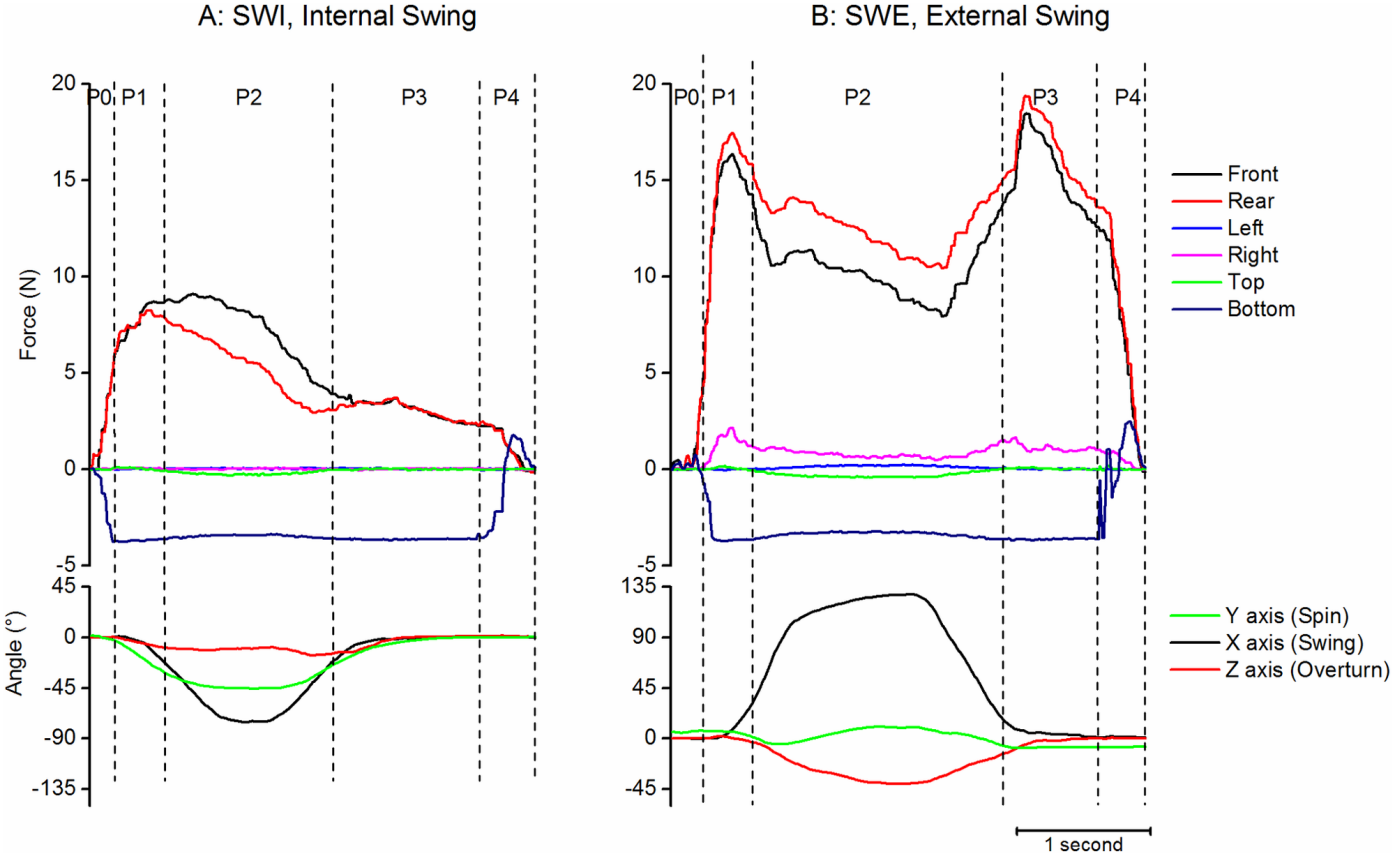
Examples of swing tasks. A: Internal Swing (pretending to drink). B: External Swing (pretending to read). Same legend as Fig 7.

Swing tasks correspond to a rotation of the object around its X axis (Fig 9). For External Swing (SWE, “pretending to read”), the force decreased during P2 by reference to P1 (Wilcoxon p<0.0001) and increased again before the deposit (Wilcoxon p<0.0001). On the rear face, the average level of force varied little during P1 to P3 phases to support the weight of the iBox (Wilcoxon p<0.02 between P1 and P2, not significant between P2 and P3).

For Internal Swing (SWI, “*pretending to drink*”), the force on the front face remained high during P2 then decreased during P3 (Wilcoxon p<0.0001). On the rear face, the force slightly decreased during P2 (Wilcoxon p<0.0001) and remained stable during P3.

As expected the force was larger on the front than on the rear face during P2 for SWI (2.3N (0.48)) and conversely larger on the rear than on the front face for SWE (2.1N (0.75)). The general level of force during P2 was higher for external than for internal swing (SWE: 10.3N (6.0) and 12.3N (6.6) for front and rear faces; SWI: 6.5N (4.4) and 4.0N (3.7), Mann Whitney p <0.0001).

The maximum rotation around X was -71.0° (25.9) for SWI and 125.2° (24.9) for SWE.

#### Overturn tasks

**Fig 10:**
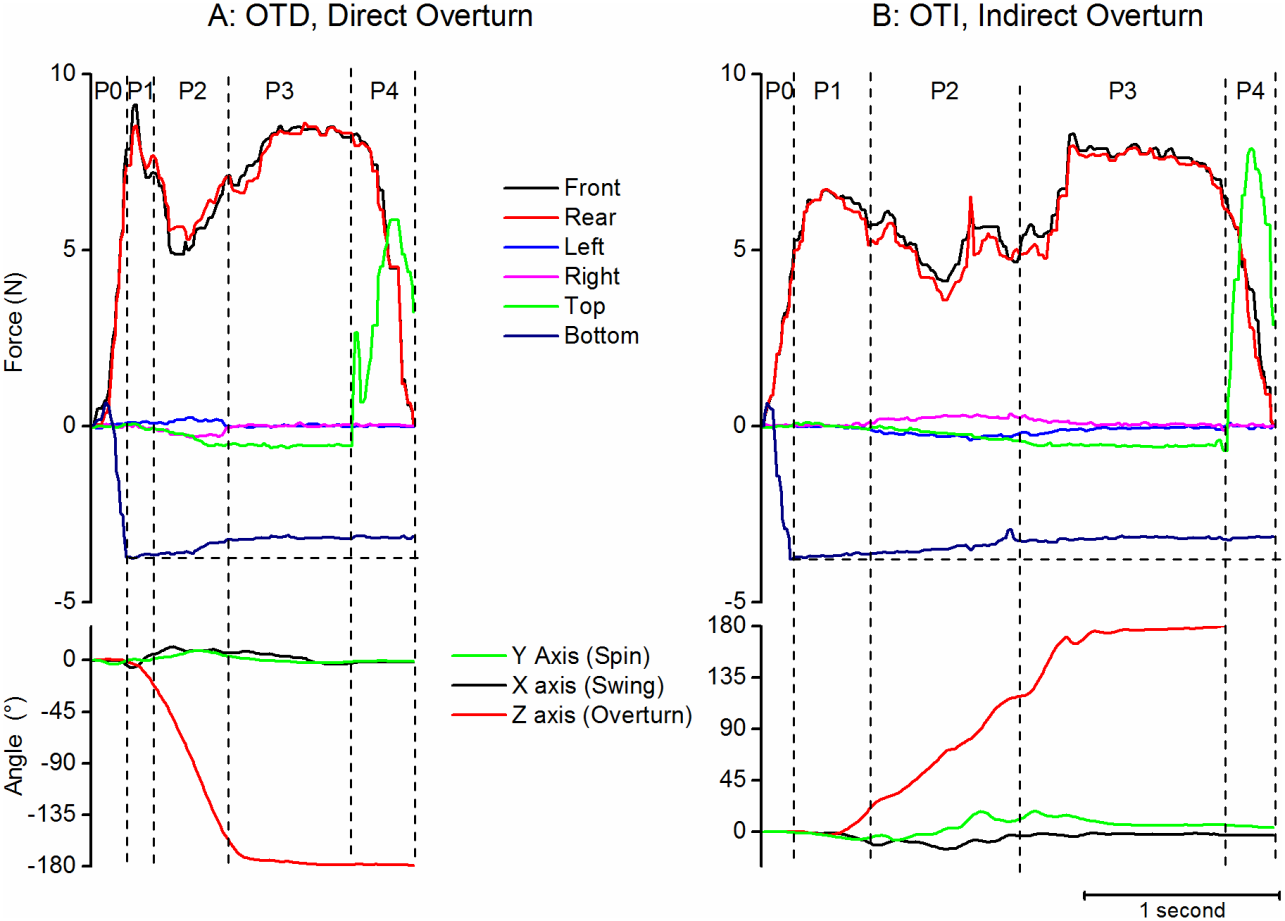
Examples of overturn tasks. A: Direct Overturn OTD. B: Indirect overturn OTI. Same legend as Fig 7.

Overturn tasks correspond to a rotation of the object around its Z axis (Fig 10).

The median forces during P2 were not different for Direct (OTD) and Indirect (OTI) Overturn (Mann Whitney, ns), they were 5.6N (2.9) and 5.3N (3.1) respectively on the front face and 5.4N (2.7) and 5.9N (3.0) respectively on the rear face. During P2, the forces had a tendency to decrease by reference to P1 during Indirect Overturn (Wilcoxon p<0.01 for the front, p<0.0001 for the rear face). The forces on the right and left faces remained close to zero during the P1-P3 phases. The median force on the front face increased during P3 by reference to P2 for Indirect overturn (Wilcoxon p<0.01).

The object was close to vertical, upside-down, at the time of deposit (rotation around Z 179.3° (3.2) and -180.2° (2.9) respectively for direct and indirect overturn).

#### Spinning tasks

**Fig 11:**
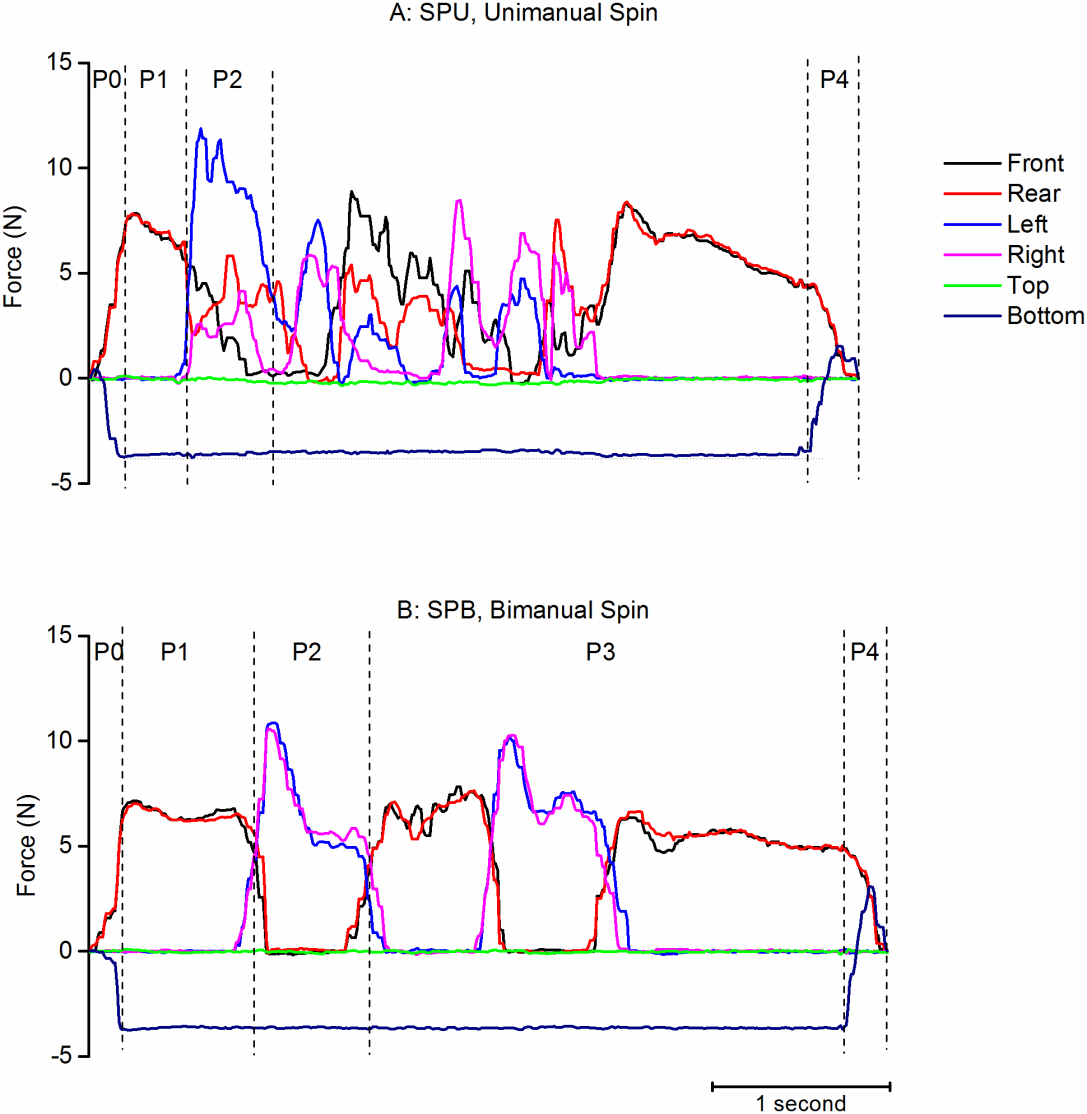
Examples of spinning tasks. A: Unimanual. B: Bimanual. Each figure represents the evolution of the time forces on the faces of the object. Phases and vertical stippled lines: same legend as Fig 4.

The spinning task was to rotate the object 360° around the Y axis (Fig 11). In contrast to other tasks, it consisted in several cycles of manipulation. For Bimanual Spinning (SPB), the explicit instruction was to produce 4 phases of 90° rotation by applying alternatively the forces on the front/rear and right/left faces of the object. This was properly executed in most of the cases, but irregularities were present in 25% of the cases (added phases for corrections of grasping or errors such as 180° rotations or grasping beginning by right/left faces). For Unimanual Spinning (SPU) the spontaneous changes of forces showed both periods with properly alternating peaks of forces and periods with force applied simultaneously on most of the faces of the object. It was not possible to count the number of phases automatically but we estimated that the task was performed with distinguishable alternative phases in 90% of the cases.

We quantified forces during the first alternation (P1 to P2). For Bimanual Spinning, the forces passed from a front/rear grasp by the right hand during P1 (4.7N (3.2) and 4.9N (3.2)) to a right/left grasp by the left hand during P2 (6.7N (3.6) and 6.9N (3.3)) with the force on the other faces close to zero. The object did not turn consistently during P1 (median less than 2°). At the end of P2, the object had turned 66.1° (22) around Y and its orientation around the vertical was very variable. For Unimanual Spinning, the trend was similar, passing from a front/rear grasp during P1 (4.6N (3.0) and 5.4N (2.5)) to a right/left grasp during P2 (5.1N (2.1) and 3.2N (1.7)). The forces on the other faces were quite small (less than 2N). The rotation of the object began during P1 (-12.7° (30), 52° (43) and 0.1° (17.3) around Y, X and Z respectively) and continued during P2 (14.0° (27.3), 49.8° (27.2) and 63.0° (19.4) around Y, X and Z respectively). The object’s inclination (relative to the vertical) allowed the weight to partially rest on the lowest face, which changed during the progression of spinning (e.g. the left face during P2 for the subject illustrated in Fig 11)

### Deposit phase (P4)

The deposit phase (P4) was characterized by the progressive loading of the bottom face (or of the previously top face after overturn tasks) while the grasping force on the front and rear faces decreased. Note that the bottom force (3.145N (0.6)) was slightly different from the weight of the object due to the extra weight (0.45N) of the top face panel (carbon+metal) of the object which was offset at the beginning of the experiment. For the same reason, the unloading of the previously bottom face was limited to -3.1N (0.4). A brisk artifact with high dynamics, probably due to vibration, was sometimes observed on the force signal of the bottom face at the onset of deposit.

The end of deposit (Table 2) was frequently characterized by an increase of force on the bottom face indicating a pushing down on the object. The frequency of apparition of “*pushes*” varied with the task from 43.1 to 75.4%. The release of the object was often followed by sub-threshold “*touches*” of the object with a frequency that varied with the task from 9.7 to 100% (Table 2).

**Table 2:** Frequency of downward *“pushes*” during P4 and “*touches*” after releasing the object.

	Pushes		Touches
tasks	%	Force	%
Precision Grip	54.2	1.5	13.9
Pinch Grip	43.1	1.4	9.7
Power Grasp	58.3	2	38.9
Top Grasp and move	62.5	1.9	37.5
Translation	75.4	1.9	34.8
External Swing	63.4	2	12.7
Internal Swing	71.4	1.9	34.9
Direct Overturn	54.2	2.3	100.0
Indirect Overturn	65.3	1.4	100.0
Unimanual Spinning	74.2	1.5	20.9
Bimanual Spinning	70.2	1.9	15.8

The grasping force measured at *td* varied significantly with the task for both the front and rear faces (Kruskal-Wallis, p<0.0001, Fig 5). This was also the case for the duration of P4 (Kruskal-Wallis, p<0.0001) and the rate of force decrease (Kruskal-Wallis, p<0.0001).

The object was almost vertical when it contacted the table at *td* (less than 1° deviation).

### Comparison of unloading and deposit phases

As shown on Fig 5, the forces applied on the front and rear faces of the object were slightly smaller at the time of deposit (4.9N (4.0)) than at the time of lifting (median of all samples 6.4N (4.3)). This was significant for all the tasks (Wilcoxon p<0.01 to 0.0001) excepted for SWE and TR. The duration of deposit was longer than that of unloading (Wilcoxon p<0.0001), except for PG, PI, SWE. The rate of force change was smaller and less variable for deposit than for unloading (Wilcoxon p<0.0001 for all the tasks excepted for SWE where p<0.05).

The frequency of “*pushes*” (Tables 1 and 2) was greater during deposit than during unloading, this was also the case for the applied forces (Wilcoxon p<0.0001 for all the tasks). The frequency of “*touches*” was greater after (9.7 to 100% of trials) than before (8.3 to 19.4% of trials) the whole action.

## Discussion

The naturalistic description of the different manipulative actions according to the time course of forces, acceleration and angles enabled by the instrumented object adds to our knowledge on human object-related actions. First, the present protocol confirms and extends previous observations on the well-known coordination of grasping and loading/unloading forces during lifting. In particular, we show that the grasping forces are also prepared as a function of subsequent object manipulation and that the object is vertical at the time of lifting and deposit (deposit has been little explored before). Second, it provides a phenomenological description of the specific adaptations of grasping forces during object rotation according to the constraints of the manipulative actions. Finally, it shows that the regulation of action sequences is prone to micro-errors particularly when the task is difficult.

### Coordination during unloading and deposit

The tight coordination between the increase of grasping forces and the unloading of the bottom force during P0 is consistent with many previous studies. It is well-known that the increase of grip force is synchronous to the increase of load force, and that both are modulated in an anticipatory way as a function of object physical properties (as reviewed in [43]). In addition, the present study shows that grip force and grip force rate are also modulated in an anticipated way (i.e. before lift-off) as a function of the subsequent task to perform.

Analyzing the deposit phase is particularly interesting as it has received little attention until now. As expected, the grasping forces were released while the bottom face was loaded. However, there were some specific differences with unloading. First, the level of grasping force was smaller than for lifting consistently with the less need to compensate the inertial effect of the weight, when the hand has only to slow down the free fall of the object. Second, there was less variation between tasks. Finally, the duration of deposit was longer than that of unloading, with smaller rate of force change. This suggests that the deposit is more stereotypic but necessitates higher spatio-temporal precision leading to increased duration, similar to Fitt’s law [44].

For all the tasks, both the unloading and the deposit were performed with the object almost vertical. Importantly, note that this was not mechanically constrained since the iBox could have been rotated around one edge of its bottom face before lift-off or, similarly, could have contacted the table by one edge at deposit after a large amount of upper-limb rotations during manipulation in some of the tasks. The vertical orientation of the object is probably an anticipated adaptation to its geometry. In addition, it confirms that the coordination of wrist and fingers for rotating the object are coordinated with great precision with the kinematics of the upper-limb to lift and descend the hand.

### Adaptation to the characteristics of the tasks

To our knowledge, the present study is the first systematic phenomenological description of force exchange during manipulation, based on Bullock’s hierarchical classification of prehensile manipulation tasks [12].

We observed that **holding** was carried out corresponding to a *“no motion /no motion at contact”* principle prioritizing the stability of grasping. The grasping forces during holding were smallest for Pinch Grip and for other tasks with similar finger configurations (“Top Grasp” and the beginning of Translation). This suggests that the opposition between the thumb and four fingers is the most convenient and economic “default” grasping configuration for the iBox as a function of its size, weight and surface. While the static friction coefficient is not proportional to the size of the contact surface, in the specific complex case of the human-finger-object contacts, it is known that it can be influenced by the surface area (larger contact surfaces increasing the friction coefficient [45]) in addition to being influenced by the normal force applied and the hydration of the skin [46]. Therefore, interpreting the normal forces amplitudes applied by the subjects is a complex exercise since the number of fingers involved in the task could possibly, through the variation of the contact area, influence the friction phenomenon, and therefore the equilibrium of forces. For example, one could speculate that grasping with the thumb and index needed more force, probably because this hand configuration was less efficient to compensate torques [5] and/or induced a minimized friction coefficient requiring higher grasp forces to compensate for object's weight [16]. The largest force was observed with the power grasp with an inconstant “dome” aspect. This suggests that the subjects voluntarily increased the grasping force above that spontaneously used for lifting, consistently with the instruction to mimic a power task [10].

The **manipulation** tasks correspond to *“prehensile task with motion related to the body”*. It is well known that when an object is displaced vertically in space, the grip force is adjusted in anticipation to the dynamical consequences of its displacement [21, 22, 47]. Several studies also demonstrated that the grip force increased in anticipation to the torsional torque needed to limit a destabilization [25, 26] or to voluntary induce a rotation of the object [26–28], with or without motion at contact [26, 27].

The present study is a tentative to generalize these findings to more ecological manipulation tasks imposing *“within hand motion* [12]*”* (motion of the object by reference to the hand due to fingers). Indeed, the tasks were designed to be difficult (Swing, Overturn) or requiring (Translation, Spinning) a contribution of the fingers.

**The Internal and External swing** were designed as tasks *“within hand motion/without motion at contact”* [12] since they imposed to maintain the iBox inclined with reference to the head according to the explicit instructions “drink” and “read”. In both cases, the grip was a combination of pinch grip and anti-gravity support by either the thumb (Internal Swing) or four fingers (External Swing). External Swing was performed with a much higher level of force than Internal Swing. This difference was not due to a different torque since the angle relative to the horizontal was grossly similar in the two situations. The relatively low level of force during Internal Swing is surprising, accounting for the difficulty to stabilize the object using only one finger that makes a relatively small surface for the gravity support [48]. The high level of force during External Swing might be due to the fact this is an uncommon action implying supination close to the joint limits. One explanation could be that External Swing disrupted the automatic correction of slipping which opposes the downward slipping of the object [34, 49]. Indeed, External Swing reversed the direction of the friction due to gravity by reference to the transversal axis of the hand, possibly perturbing automatic corrections and thus bringing the subject to perform stronger grips as in situations with decreased sensation [50].

The **Translation** task imposed “*within hand motion/motion at contact*” [12]. Translation was performed by a brisk (but not quantified in the present study) lowering of the force on the front/rear faces while the force increased temporarily on the right/left faces.

**The Overturn** tasks could theoretically be performed according to two strategies: “*without in-hand motion*” (the task is performed with a movement of the whole upper-limb) or “*within hand motion/motion at contact”* (the rotation can be facilitated by grasping the objects below the level of the center of mass, so that a small rotation is enough to destabilize the object, the grip force acting to break the descent). The instruction specified to avoid global movements that in addition would have been less comfortable [39] and more energetically costly. For Indirect Overturn the subjects grasped the object by the left side with a hyper-flexed and pronated wrist (see Fig 2) so that supination was enough to rotate the object to its final position, likely without motion at contact. In contrast, for the Direct Overturn the subjects probably had to allow some motion at contact. However, Direct and Indirect overturns were performed with a similar level of force and we could not automatically distinguish the precise strategies used by the subjects.

The **Unimanual Spinning** task was the most difficult to execute, imposing both complex *“within hand motion”* and *“motion at contact”*. It was performed by sharing alternatively grip forces between the front/rear and the right/left faces and by inclining the object from the vertical so that the weight was partially supported in the palm (i.e. combining “prehensile” and “non-prehensile” hand configurations [3]). This shows that grasping forces can be smoothly combined to support gravity during manipulation. The grip forces were relatively low during the first phase of Spinning probably to allow more flexibility in the contact between fingers and object.

For **Bimanual Spinning**, the alternation of grip forces on the front/rear and right/left faces was very clear and the object was kept almost vertical. Most previous studies on bimanual organization of natural actions were observations performed in the context of infant development [51] or evolution of primates [52]. There are still few quantitative studies on bimanual cooperative actions in adult humans [53]. Further studies are needed to precise the role of both hands in cooperative bimanual activities.

### Regulation of action sequence

The tasks consisted in several sub-goals: grasp, lift, hold or manipulate, deposit and release. The present study confirmed that grasping and the first phase of lifting (unloading) were performed synchronously, as usually observed in the literature [54]. The present study affords additional information that the object was lifted and deposed vertically, demonstrating a sequential organization of unloading and manipulation. However, the rotations imposed by the task began during the phase of acceleration of the lifting movement (Fig 6) showing that the sub-goals lifting and rotation were smoothly combined.

The phenomena of “*touches*” and “*pushes*” can be considered as micro-errors occurring in the action sequence (i.e. inappropriate elements of movement automatically detected and corrected before the error was completed [55, 56]). Despite the decreased force rate for deposit, suggesting increased caution, they were more frequent at deposit and their occurrence was clearly dependent of the task. The “*touches*” were constantly observed after object Overturn, perhaps reflecting a difficulty of hand clearance. More generally, motor errors were more frequent for the tasks implying more force (Power Grasp) or more difficulty (Overturn, External Swing, Spinning). Manipulation of the iBox could be a mean for a better detection and understanding of those subtle errors in cognitive and sensorimotor action regulation [57]. Another possible interpretation could be that “touches” and “pushes” before grasping represent a groping behaviour used to explore the affordances of the object and support surface before the execution of the action. However, their functional role is unlikely in the present study since the initial conditions (same position of the same object) were constant across tasks and since the sensory information (in particular vision) were not altered”. The functional role of “*touches*” and “*pushes*” after manipulation during or after deposit is still less likely. Further studies are needed to precise these aspects in healthy subjects and in a variety of pathological situations.

## Conclusion: Limitations and perspectives

The protocol proposed here combined several manipulation tasks of graded difficulty allowing the quantitative analysis of force exchanges during manipulation of a novel instrumented object. As expected, we confirmed the fine regulation of grasping and unloading forces during lifting, the economic grasp being an opposition between thumb and four fingers. In addition, we showed that the grasping forces were finely regulated according to the specific constraints of each task. When the task imposed a rotation, it started just after lift-off in parallel with the lifting movement and was completed just before deposit so that the object is vertical at the time to contact. However, with increasing task difficulties, the course of action sequence was disturbed by micro-errors evidenced by extra “touches” or “pushes” on the object.

The main limitation of the present study is that the instrumentation gave only indirect data since it did not quantify the object motion relatively to the hand nor the contact of individual fingers with the object. Therefore, we could not identify completely the alternative grasping strategies used by the subjects: we cannot separate “within hand motion” due to the fingers from “motion relative to the body” due to wrist or proximal joints motion nor quantify “motion at contact”. Another limitation is that the iBox measures normal forces, so that variations in the friction at contact induce modulations of the measured magnitude of force. On the other hand, this made the analysis tractable with a relatively simple and light object, and avoided an explosion of the recorded data.

Future studies should take into account these limitations. The instruction given to the subject should more clearly specify the configuration of the fingers for grasping and whether motion at contact is allowed. The device could be improved by a modification of its structure (a different disposition of reasonably priced force sensors, better IMU, pressure sensitive sensors to detect finger position…). More sophisticated signal analysis method should be developed for the analysis of the acceleration signal to better precise the timing of action sequence and for the automatic classification of tasks from multimodal signals. However, measuring torques and the precise fingers position on the device would imply using 6DoF force sensors and sensors to detect the position of each finger, which would result in a complex and likely heavy object. Furthermore, this would make the data analysis more complex and might not give much more insight on manipulation strategies.

Importantly, the present protocol with tasks of graded difficulties seems to be suitable for the clinical assessment of grasping in patients with different levels of impairment. Patients with a severe motor impairment can take the object with gross whole hand grasping or alternative grasping strategies [38] while patients with a mild disorder may exhibit specific impairments when performing complex manipulation tasks such as spinning. Further studies with more various instrumented objects are also needed for specific tasks (e.g. key grip and door opening [58]).

## Abbreviations

OTD: Direct overturn
OTI: Indirect overturn
P0: unloading phase, between *tg* and *tl*
P1: lifting phase, between *tl* and *tp*
P2: holding or manipulation phase, between *tp* and *tf*
P3: preparation to deposit, between *tf* and *td*
P4: deposit phase, between *td* and *te*
PG: Lifting with precision grip
PI: Lifting with pinch grip
PO: Lifting with power grasp
SPB: Bimanual spinning
SPU: Unimanual spinning
SWE: External swing, “pretending to read” on the bottom face
SWI: Internal swing, “pretending to drink”
*td*: end of lifting
*te*: end of grasping
*tf*: end of the holding or manipulation period
*tg*: onset of grasping
*tl*: onset of lifting
TO: Lifting from the top and move
*tp*: beginning of the holding or manipulation period
TR: Lifting with precision grip then translating to power grasp
